# Bimanual Coordination Learning with Different Augmented Feedback Modalities and Information Types

**DOI:** 10.1371/journal.pone.0149221

**Published:** 2016-02-19

**Authors:** Shiau-Chuen Chiou, Erik Chihhung Chang

**Affiliations:** Institute of Cognitive Neuroscience, National Central University, Taoyuan City, Taiwan; University of California, Merced, UNITED STATES

## Abstract

Previous studies have shown that bimanual coordination learning is more resistant to the removal of augmented feedback when acquired with auditory than with visual channel. However, it is unclear whether this differential “guidance effect” between feedback modalities is due to enhanced sensorimotor integration via the non-dominant auditory channel or strengthened linkage to kinesthetic information under rhythmic input. The current study aimed to examine how modalities (visual vs. auditory) and information types (continuous visuospatial vs. discrete rhythmic) of concurrent augmented feedback influence bimanual coordination learning. Participants either learned a 90°-out-of-phase pattern for three consecutive days with Lissajous feedback indicating the integrated position of both arms, or with visual or auditory rhythmic feedback reflecting the relative timing of the movement. The results showed diverse performance change after practice when the feedback was removed between Lissajous and the other two rhythmic groups, indicating that the guidance effect may be modulated by the type of information provided during practice. Moreover, significant performance improvement in the dual-task condition where the irregular rhythm counting task was applied as a secondary task also suggested that lower involvement of conscious control may result in better performance in bimanual coordination.

## Introduction

When acquiring a novel motor skill, augmented feedback such as the usage of mirrors in a dance studio or a haptic guidance during physical therapy is usually provided to facilitate error correction and to help learners acquire basic knowledge about the movement. The term “augmented feedback”, also known as “extrinsic feedback”, refers to externally presented information about the outcome or the execution process of a movement. Although it can often lead to significant improvement in the acquisition phase, continuously using the augmented feedback seems to result in dependence easily as revealed by performance deterioration upon its removal [1, 2].

The nature of augmented feedback in motor learning has elicited some discussions in the literature. Some researchers suggest that motor learning involves a shift from closed-loop to open-loop control [3, 4]; that is, through a repetitive feedback-based error correction process, one can gradually develop an internal movement representation or “motor program”, which then assists learners to execute a movement independently. Therefore, as learners become increasingly skillful, their dependence on the feedback should decrease [5]. However, other empirical observations support an alternative view that the dependence on the feedback may not decrease as practice proceeds. As shown in Proteau et al. [6], participants experiencing greater amount of practice suffered more severe feedback dependence in a manual aiming task than those who had less practice. The phenomenon leads to the so-called “specificity-of-learning hypothesis” [7] which asserts that, instead of forming an independent motor representation, motor learning involves the development of a complex “sensorimotor reference mechanism” which consists of integrated information from both central control process and sensory feedback. Therefore, motor learning is specific to the sources of sensory information which are available during acquisition, and motor performance is optimal if the availability of the feedback information is similar between the practice and the test phases.

From another point of view, the “guidance hypothesis” suggests that the feedback during acquisition acts as a “guidance” to enhance performance, while excluding the engagement of other information processing activities which are essential to successful retention, such as more efficient error detection and correction processes or the development of an independent motor representation. Therefore, performance deteriorates when the feedback was removed, which is coined as the “guidance effect” [2, 8, 9].

The feedback-dependent decrement of transfer has been widely observed in motor learning studies including bimanual coordination, especially when the concurrent visual feedback was used. In Kovacs et al. [10], participants learned a 90°-out-of-phase bimanual coordination pattern (i.e., one arm leads the other by a quarter-cycle but both arms move in the same frequency) with a concurrent Lissajous feedback. The Lissajous feedback integrates the displacement information of both effectors by representing it on the abscissa and ordinate of a single cursor plot, respectively, and has been demonstrated to be effective to enhance performance in bimanual coordination [11–14]. The results in Kovacs et al. [10] showed that the participants performed the coordination pattern quite successfully within only 5 minutes of practice, but the removal of the feedback led to dramatic performance deterioration.

According to the guidance hypothesis, a reduced feedback schedule would allow participants to process more intrinsic information and to develop an independent motor representation which they can rely on when the feedback is removed. Kovacs et al. [15] successfully showed that a reduced feedback schedule can help participants overcome the guidance effect when learning a bimanual coordination task with Lissajous feedback. Other studies further demonstrated that the guidance effect is not simply related to how often the feedback is provided, but also how the feedback is displayed. In Buchanan et al. [16], they manipulated the display format of the Lissajous feedback (with the cursor “superimposed” on the Lissajous template or with the cursor presented in a “separated” window) and showed that a 100% feedback presentation schedule can still be beneficial when the Lissajous plot and the Lissajous template were separated. The study illustrated that more attentional efforts directed to the proprioceptive feedback and more emphasis on the use of intrinsic processes may enhance the development of motor representation and benefit long-term retention.

Moreover, in Ronsse et al. [17], participants learned the 90°-out-of-phase bimanual coordination pattern with either visual or auditory feedback. For the visual group, concurrent Lissajous feedback was provided, and for the auditory group, low-pitch and high-pitch tones were played back when participants’ left and right hands reached the reversal points, respectively. This auditory feedback can be viewed as a unified temporal structure as it integrated the motor information of both effectors by a series of rhythm, and when the task was performed correctly, it would result in four equally spaced tones for each movement cycle. In line with previous findings, learning with visual (Lissajous) feedback led to significant performance deterioration after feedback removal, while learning with auditory (rhythmic) feedback was more perseverant. The corresponding functional magnetic resonance imaging results also showed that, during the practice phase, brain activation increased in sensory-specific areas for visual group, suggesting a dependence on the augmented feedback; whereas the brain activation decreased for the auditory group, specifically in areas associated with cognitive and sensory monitoring of motor task performance, supporting the idea that as practice proceeds, an independent control strategy gradually develops and the reliance on the feedback decreases.

Ostensibly, Ronsse et al. [17] demonstrated a modality-dependent behavior in bimanual coordination learning with augmented feedback; that is, learning with auditory feedback had better retention after feedback removal than learning with visual feedback. However, as they provided continuous Lissajous feedback to the visual group but discrete rhythmic feedback to the auditory group, feedback modality (visual vs. auditory) was confounded with feedback information (continuous visuospatial vs. discrete rhythmic), and thus the results may not be solely attributed to the modality difference between groups, but also the difference in feedback information.

In the literature, “visual dominance” is a widespread phenomenon which means that the visual input tends to gain priority of processing and resources in perception or memory [18]. As visual dominance is so pervasive in human information processing, it is likely that the stronger guidance effect accompanying the visual augmented feedback is due to the dominant visual information blocking the processing of other “non-dominant” sensory sources, especially the proprioception which is essential for establishing a permanent, genuine motor representation. On the contrary, auditory feedback is less dominant and thus may allow superior sensorimotor integration and deeper processing of the proprioceptive information.

However, an alternative theoretical perspective on the differential guidance effect is that, the better retention performance observed in the auditory group may simply result from stronger linkage between the rhythm information and the human kinesthesis, or a better control strategy developed under the discrete feedback condition compared with the continuous one. Thus, instead of being “modality-dependent”, the guidance effect may be modulated by the structural feature of the feedback information.

In sum, it is unclear whether previous report of differential retention performance between learning with visual and learning with auditory augmented feedback [17] was indeed modality-dependent, or actually due to different structural feature of the feedback information. The aim of the current study is thus to investigate how modalities (visual vs. auditory) and information types (continuous visuospatial vs. discrete rhythmic) of concurrent augmented feedback influence bimanual coordination learning.

In the present experiment, three groups of participants learned a 90°-out-of-phase bimanual coordination pattern with (1) Lissajous feedback (Lissajous group) which indicates the integrated position of both arms, (2) visual rhythmic feedback (Color group) which reflects the relative timing of the arms’ movement by discrete visual information, or (3) auditory rhythmic feedback (Tone group) which reflects the relative timing of the arms’ movement by discrete auditory information. The design of the rhythmic feedback provided to the last two groups were similar to that imposed in Ronsse et al. [17] but displayed in either visual or auditory format. Each group of participants performed the motor task in a practice phase lasting three consecutive days and in a test phase which included two blocks of no-feedback transfer (one was applied right after the practice phase on day 3 and the other was applied as 24-hour retention on day4). By adding an additional visual rhythmic group (Color group), we can examine whether “visual dominance” is a determinant factor of inducing guidance effect by comparing the retention performance between Lissajous and Color groups (as both feedback are displayed in visual format but with different structural features); we can also test how bimanual coordination learning is influenced by the structural feature of the feedback information by comparing the performance between Color and Tone groups (as both feedback are in rhythmic format but displayed in different modalities).

Following the rationale of the “visual dominance” account for the guidance effect, one would predict greater performance deterioration after feedback removal in both the Lissajous and the Color groups than in the Tone group because visual feedback occupies too much mental resources during learning, leaving insufficient resources for processing other sensory information, including the proprioception. Alternatively, following the logic of the “structural feature” account, the prediction would be that the performance of the Color group and the Tone group will be more resistant to the feedback removal than that of the Lissajous group because the continuous visuospatial information such as Lissajous figure may strengthen the dependence on the augmented feedback, while the discrete rhythm information benefits long-term retention.

In addition, according to the challenge point framework [19], learning is related to the information available and interpretable during practice. Thus, as for participants’ performance during practice phase, Lissajous group is predicted to have the lowest error because the participants have continuous access to the phase relationship of the two effectors. The Tone group is also predicted to have slightly better performance than the Color group given that higher temporal resolution of the auditory system (which is at millisecond level [20–23] compared to tens of millisecond level of the visual system [24]) may facilitate the processing and the interpretation of the rhythmic feedback information.

Furthermore, in order to examine the level of conscious control and the potential strategies adopted in the motor execution process, a dual-task interference session with irregular rhythm counting as a secondary task was included right after the retention test on day 4. In a dual-task paradigm, a secondary task is expected to interfere with the primary task if the two share similar skill sets or demand for the same resource. Therefore, if participants adopted a more conscious control strategy to reproduce the movement in the current experiment (especially when rhythm recall was involved), relatively severe performance deterioration is anticipated in dual-task condition because the cognitive resources for consciously controlling the movement was reduced; alternatively, participants’ performance should remain unchanged if the movement was performed automatically with minimal demand of cognitive resources.

## Method

### Ethics statement

The experiment was conducted in accordance with the ethical principles stated within the declaration of Helsinki (1964) and was approved by the Research Ethics Committee in the National Taiwan University. The participants signed informed consent prior to the experiment and were paid NT$ 500 (approximately US$ 16) for their participation after the completion of all experimental sessions.

### Participants

Twenty-two adult participants were recruited to participate in the experiment. All participants had no prior experience to the experimental task, and none of them had ever experienced intensive musical or athletic training. They were randomly assigned to Lissajous (*n* = 8, mean age = 22.50, *SD* = 3.07, 6 females, 2 males), Color (*n* = 7, mean age = 22.29, *SD* = 3.09, 3 females, 4 males), and Tone (*n* = 7, mean age = 21.71, *SD* = 1.70, 4 females, 3 males) groups. Note that twenty-eight participants were originally recruited for the experiment. However, six of them (three in Color and three in Tone groups) failed to achieve required movement proficiency by the end of the three-day practice phase and thus were excluded from further analyses.

### Apparatus

**Fig 1:**
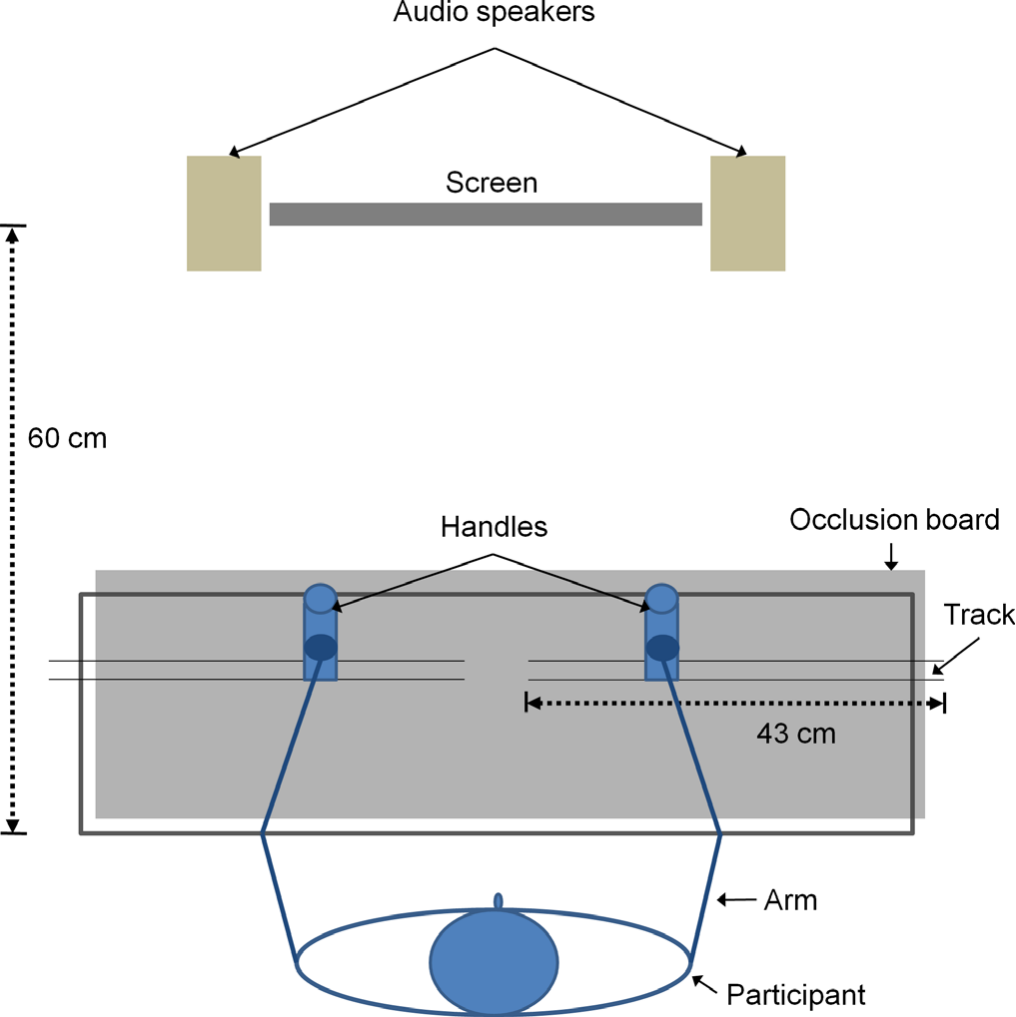
Illustration of the participant and apparatus.

Participants sat in front of a wooden table (height = 68 cm) where they could comfortably rest and extend their forearms. Two 43-cm long and 3-cm wide plastic tracks were fixed on the table and parallel to the participant’s medial-lateral axis. The participant held two handles which could be moved along the tracks, restricting the arm movement to the medial-lateral direction. Attached to each handle was a sensor of a magnetic motion capture system (3DGuidance trakSTAR^TM^, Ascension Technology Corporation, Shelburne, Vermont, USA), respectively, whose 3D coordinates were digitized and registered with a measurement rate of 80 Hz. By using the data, the “real time” augmented feedback could be provided to the participant with minimal delay limited by the screen refresh rate (60 Hz) and the time lag in the motion capture system. Visual feedback was displayed on a LCD screen at a distance of 60 cm in front of the participant, and auditory feedback was played via two audio speakers located symmetrically beside the screen. Both hands and forearms were occluded from the participant’s view throughout the experimental sessions (Fig 1). Experimental flow and data analysis were programmed in Python, and stimuli presentation was implemented with the Psychopy toolbox [25, 26].

### Tasks and groups

**Fig 2:**
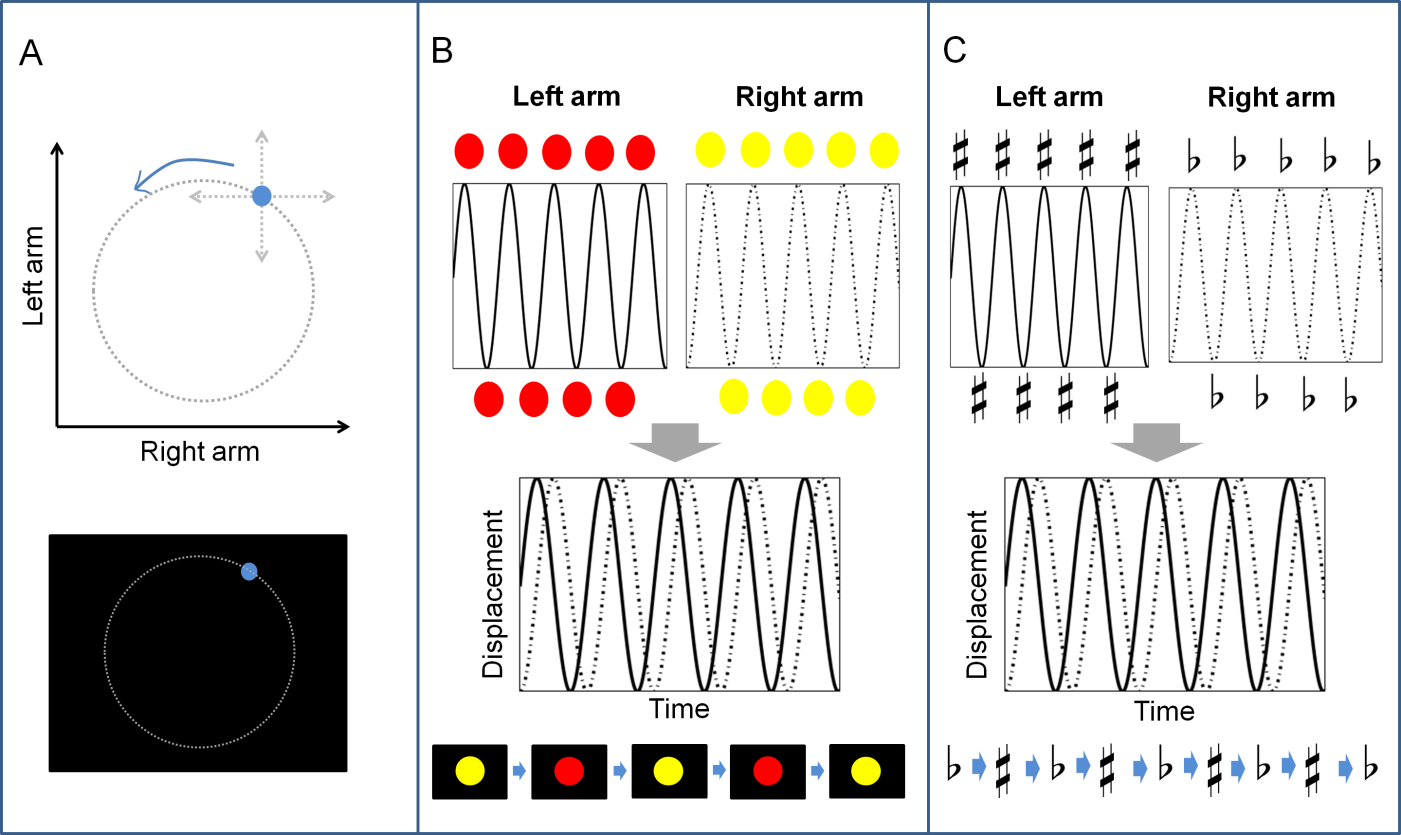
Illustration of different augmented feedback. (A) Lissajous (continuous visuospatial) feedback. Note that the dotted circle configuration is for clarity of illustration and was not seen by the participant. (B) Color (discrete visual rhythmic) feedback. (C) Tone (discrete auditory rhythmic) feedback.

During the experiment, participants learned to move their arms along the medial-lateral direction cyclically with one arm leading the other by a quarter-cycle, namely a 90°-out-of-phase (*ϕ* = 90°) coordination pattern. Compared with in-phase (*ϕ* = 0°) and anti-phase (*ϕ* = 180°) coordination patterns, stable and consistent performance of the 90°-out-of-phase coordination pattern requires intensive practice [11, 14]. Each participant was assigned to one of the three groups receiving different types of augmented feedback during practice. (1) Lissajous group. A cyan cursor (0.6 degree in diameter) of which abscissa and ordinate representing the positions of the arms on the track was presented continuously throughout a learning trial. Specifically, the participant’s right-arm movement along the track would move the cursor along the horizontal axis (x coordinate), and the left-arm movement would move the cursor along the vertical axis (y coordinate). The trajectory of the cursor motion can be described by the following equation representing complex harmonic motion known as Lissajous curves [27]:

x=Asin(at+δ),y=Bsin(bt) (1)

where *a* and *b* indicate angular velocity, *δ* indicates phase difference, and *A* and *B* are scaling factors. According to the Eq (1), when moving the arms with the same frequency (*a / b* = 1) and amplitude (*A = B*) but with the phase difference of 90 degrees (*δ* = 90°), the cursor would move along a circular trajectory (Fig 2A). (2) Color group. In the cyclical medial-lateral arm movements carried out by the participant, there were two reversal points in a cycle for each arm. When the participant’s right arm reached the reversal points, a yellow disc (4 degrees in diameter) would flash in the center of the screen, and when the left arm reached the reversal points, a red disc would flash. The discs were presented for 150 ms after each onset. If the participant coordinated the bimanual movement correctly, they would see a rhythmic, equally spaced alternation of the yellow and the red discs (Fig 2B). (3) Tone group. Similar to the Color group, the reversal points of the movement would be detected, but it elicited feedback in the form of low-pitch (700 Hz) and high-pitch (1040 Hz) tones for right and left arms, respectively. The tone would last for 150 ms after its onset. Therefore, correct coordination of the arm movements would lead to rhythmic, equally spaced alternation of the low-pitch and the high-pitch tones (Fig 2C). By providing concurrent augmented feedback as indicated above, participants received information about their performance instantly and made on-line adjustments accordingly.

### Procedure

The experimenter first explained the motor task by briefly showing three 5-second animation clips, in which there were two moving bars aligned as two inverted pendulum, moving first in the in-phase mode, then the anti-phase mode, and finally the 90°-out-of-phase mode (see S1–S3 Videos). These animations helped the participants to get a general idea about the targeted movement without providing sufficient details which might allow them to learn the task merely by watching. In addition, using the animation clips avoided too much verbal instruction which may bias participants’ learning process away from their natural course. The experimenter then explained how the augmented feedback works. Participants could play with the feedback in their own pace for one to two minutes to get familiar with the relationship between the arm movement and the feedback. Note that the scale used in the movement-cursor mapping in the Lissajous group and the reversal points used in the Color and the Tone groups depended on the movement range of each participant, which was calibrated on their in-phase movement performed before the practice began. One demo practice trial (see below for more details) would then be presented, while additional ones would be given only if the participants were still not clear about the task.

The practice phase and test phase constitute the main parts of the experiment to examine bimanual coordination learning with augmented feedback and the retention performance when the feedback was removed. An additional dual-task interference session was also administered at the end of the experiment to further investigate the control strategy that the participants adopted and the motor representation that was developed during practice.

#### a. Practice phase

**Fig 3:**
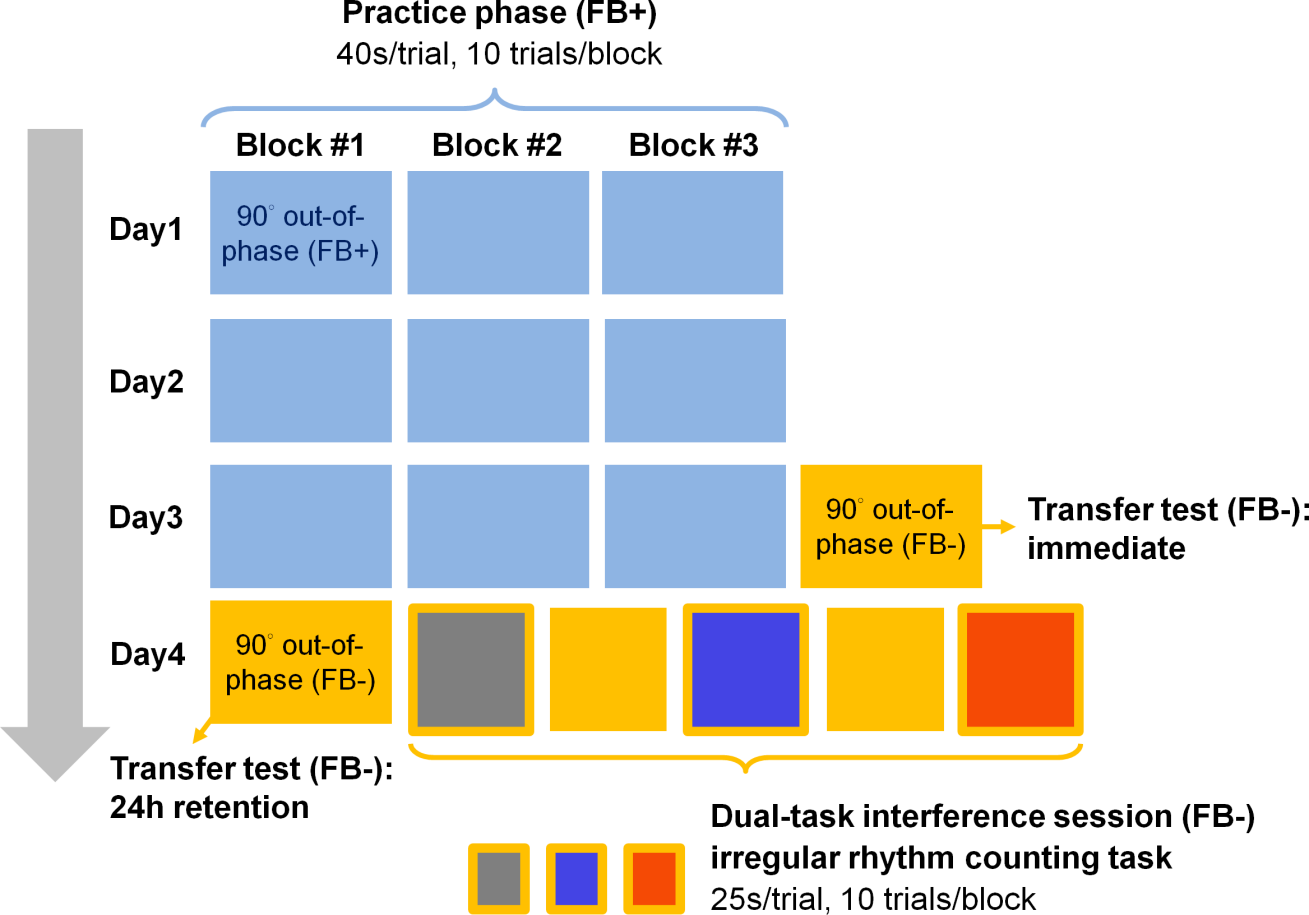
Experimental protocol. In the practice phase, participants practiced for three blocks per day (10 trials/block, 40 s/trial) with augmented feedback. After practicing for three consecutive days, two sessions of the no-feedback transfer test were applied on day 3 (immediate test) and day 4 (24-hour retention test), respectively. Finally, in the dual-task interference session, participants performed three different types of irregular rhythm counting tasks (each type for one block, 10 trials/block, 25 s/trial) concurrently with the motor task. Two “single-task” blocks (motor task only without augmented feedback) were interleaved between the interference blocks.

In the practice phase, participants learned to perform the 90°-out-of-phase coordination pattern with the concurrent augmented feedback for three consecutive days. They practiced for three blocks per day, and each block contained 10 trials (Fig 3). Within each trial, participants would first see a preparation notice, and they were requested to set their forearms at the preparation positions before a new trial started. Preparation positions were the middle points of the movement paths, approximately aligned with the width of the shoulders. After the preparation notice, participants would observe the ideal feedback pattern for 10 seconds, demonstrating how the feedback looked or heard if performing the movement correctly (Fig 2A–2C. Also see “Tasks and groups” in Method section for more details about the ideal feedback pattern presented in each group. Movement frequency was set at 0.8 Hz in all three conditions, which most participants in a pilot experiment can use the feedback information without difficulties and gain steady improvements during practice). Following the demonstration, participants practiced for 40 seconds. They were instructed to reproduce the ideal feedback pattern as best as they could by the arm movements. The display of the ideal feedback pattern at the beginning of each trial was to strengthen the mental image of the movement target and thus facilitate error correction process. In addition, before starting the practice each day, participants were reminded again of the guidelines about the task, including “try to move the arms continuously and smoothly without any sudden acceleration or deceleration”, “try to reproduce the trajectory (or the rhythm) as similar as the ideal one”, and also, “remember that the arm movement can control the cursor (or the appearance of the discs and the tones)”. One practice block lasted approximately 10 minutes. Participants took a short break between blocks, usually for one to three minutes as they desired.

#### b. Test phase

After practicing for three consecutive days, participants were tested unexpectedly on a block of no-feedback transfer test right after the last block of practice on day 3. The 10-second display of the ideal cursor trajectory or rhythmic pattern at the beginning of the practice trial was also replaced with a 10-second blank. Other procedures remained the same as practice trials. An additional block of the no-feedback transfer test was administered again on day 4 to further examine participants’ retention performance after 24 hours.

#### c. Dual-task interference session

Participants performed the following three different types of irregular rhythm counting tasks concurrently with their acquired motor task (augmented feedback was not provided) in the dual-task interference session: (1) visual rhythm–continuous (VR-C), (2) visual rhythm–discrete (VR-D), and (3) auditory rhythm–discrete (AR-D). Each type of counting corresponded to one of the three practice conditions in order to check if there was any interaction between the way the irregular rhythm stimuli presented and the type of augmented feedback the participants used during practice. As the core manipulation here was the interference from rhythm counting, one would not expect to observe interactions between types of irregular rhythm and augmented feedback, which implies potential interference due to similarity between them. For this purpose, the stimulus features (color and pitch) of the counting stimuli were made distinct from the practice phase to avoid confusion.

**Fig 4:**
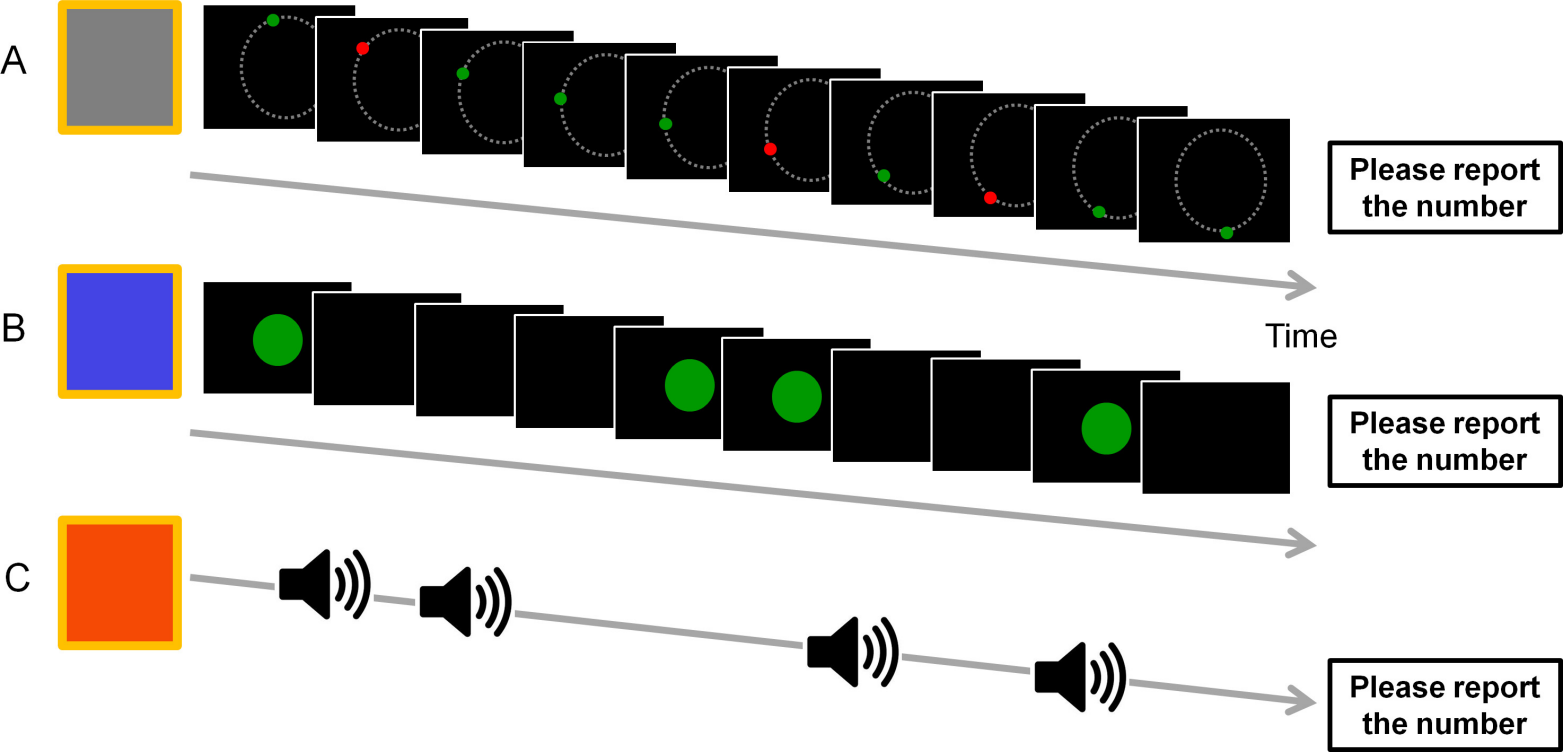
Illustration of the irregular rhythm counting task. (A) Visual rhythm–continuous (VR-C). (B) Visual rhythm–discrete (VR-D). (C) Auditory rhythm–discrete (AR-D).

In the VR-C condition, a green dot (0.6 degree in diameter) moved along a circular trajectory at a speed of 0.5 Hz. The movement of the dot was similar to the ideal pattern provided to the Lissajous group during practice except for its color and speed. The green dot turned red for 150 ms in an irregular rhythm. Participants were instructed to count the number of color changes during a 25-second period and orally report the number of changes at the end of a trial. In the VR-D condition, a green disc (4 degrees in diameter) flashed irregularly at the center of the screen (lasting for 150 ms), and the participants were instructed to count the appearance of the disc and report the number of appearance at the end of a trial. In the AR-D condition, there was a series of short beep sounds displayed in an irregular manner. Each sound lasted for 150 ms at the pitch of 930 Hz, different from the ones used in the Tone condition. Participants were instructed to count the number of the beep sounds and report at the end of a trial (Fig 4).

The timing of presenting the irregular stimulus was determined by shuffling 7 different inter-stimulus intervals: 0.5 s, 0.75 s, 1 s, 1.25 s, 1.5 s, 2 s, and 2.5 s in all three conditions. Before each interference block began, participants would first practice the irregular rhythm counting task without any arm movements, and only if they performed correctly for three consecutive trials could they start to perform in the dual-task condition. Each interference block consisted of 10 trials. Within each trial, participants would first see a preparation notice followed by a cue tone, signaling to initiate the movement. Irregular rhythm would then be displayed. Participants counted the number at the same time as they performed the coordination task. After 25 seconds, a cue tone was played again indicating to terminate the movement and to report the number to the experimenter. Note that participants were instructed to prioritize counting accuracy as their primary task goal.

Participants encountered the three different interference blocks of counting tasks in a randomized order to balance potential serial order effect. Moreover, because every participant experienced all three interference conditions, in order to “washout” the motor memory from the previous interference block and to reduce the potential anterograde interference, two additional “single-task” blocks (5 trials in a block, 40-second movement in each trial) during which participants performed the motor task (no feedback) without any interference were interleaved between the interference blocks (Fig 3).

### Dependent measures and data analysis

The 3D-coordinates from the first five seconds of each trial were discarded to eliminate records of the movement initiation when the movement trajectory was typically highly variable. Displacements of participants’ arm movement were low-pass filtered with a fourth-order Butterworth filter. Cut-off frequencies were determined adaptively for each trial based on residual analysis proposed by Winter [28], and a spline fit was then applied to realign data across groups with data frequency of 60 Hz.

Participants’ coordination performance of the motor task was evaluated by comparing the continuous phase difference between right and left arms with the task goal of 90 degrees. Prior to analysis, displacement and velocity data were normalized between -1 and 1 by first subtracting mean of each data series from each data point to center all the data around zero, and then by rescaling the data via dividing positive and negative amplitudes with the maximum positive or negative amplitudes. The phase angle (*θ*_*i*_) for each arm (*i = right*, *left*) at each time point was then computed by using the following formula adapted from Kelso et al. [29]:

$$\theta_{i}=\tan^{-1}\left[(\Delta X_{i}/\Delta t)/X_{i}\right]$$ (2)

where *X*_i_ represents normalized displacement data of the arms and Δ*X*_i_ / Δ*t* represents normalized velocity. The continuous relative phase was calculated following circular statistics method based on Euler’s formula:

$$e^{i\theta }=\cos\theta +i\;\sin\theta$$ (3)

where relative phase (*ϕ*) defined as phase difference between right and left arms, *ϕ = θ*_*right*_*—θ*_*left*_, can be derived by computing the ratio *e*^*iθright*^ */ e*^*iθleft*^. Since the leading relationship between the arms was not concerned in the current study, the valence information was dropped from the following analysis.

To compare participants’ performance with the task goal of 90° phase difference, we calculated root mean square error (RMSE) of the relative phase as a key performance indicator. The RMSE of each trial (with *n* data points) was calculated by the following formula:

$$RMSE=\sqrt{\frac{1}{n}\sum_{i=1}^{n}{\left(\phi_{i}-90{}^{\circ}\right)}^{2}},i=1,2,\ldots ,n$$ (4)

Lower RMSE indicated better achievement of the task goal of maintaining 90° phase difference between the arms during the movement.

## Results

Statistical threshold of Type I error was set at *α* = .05. Eta squares (*η*^2^) were reported to indicate effect size. *Post hoc* analyses were conducted using Bonferroni correction.

### Performance in practice phase

**Fig 5:**
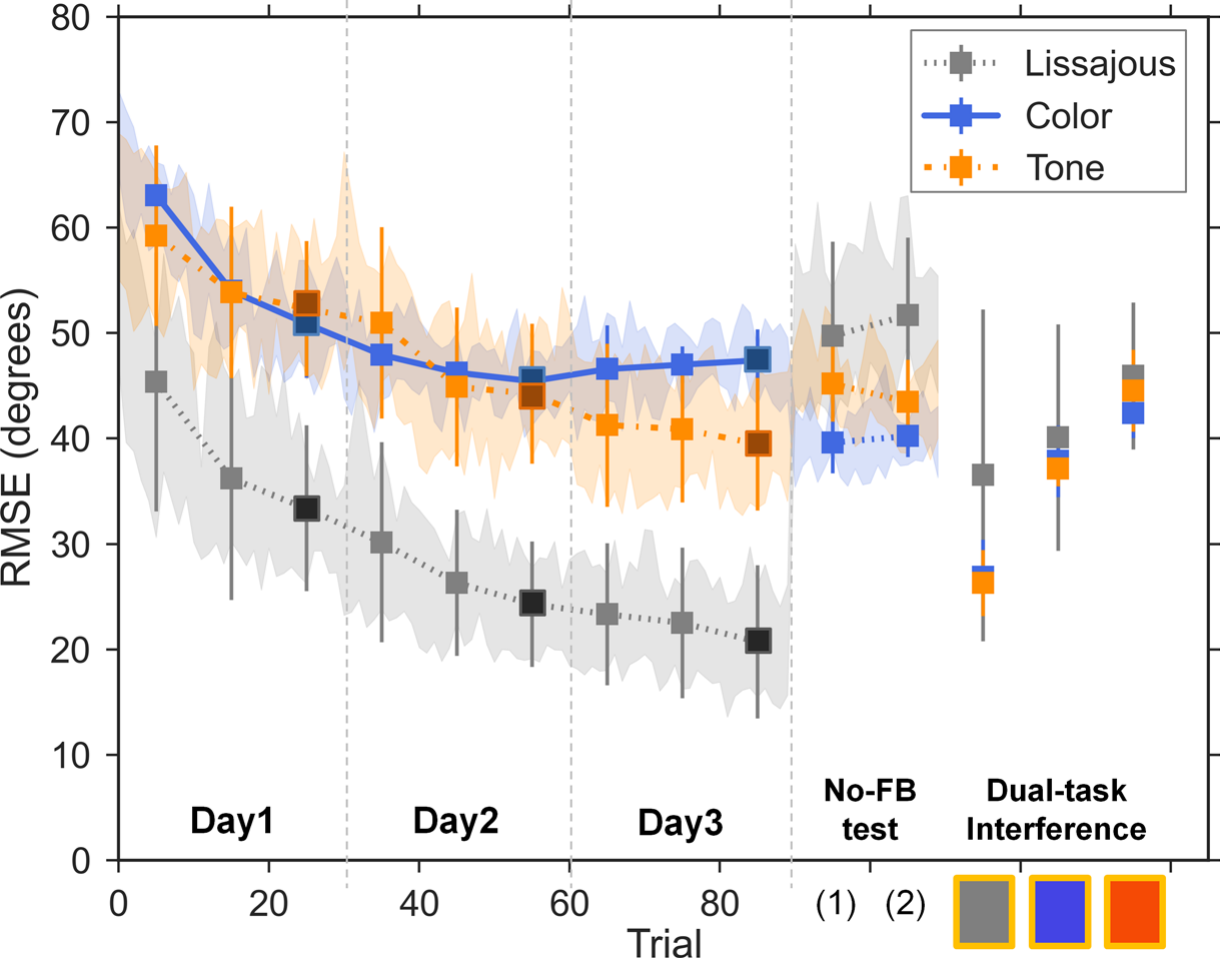
Motor performance in the practice phase, the test phase, and the dual-task interference session. Square markers represent averaged RMSE of individual blocks in each group, with the last block of each day filled in dark colors. Error bars indicate standard deviation of each block and shaded areas indicate 95% confidence interval of each trial. Two sessions in the no-feedback transfer test (No-FB) are: (1) immediate test on day 3 and (2) 24-hour retention test on day 4.

RMSE of the relative phase across practice are illustrated in Fig 5. A three-way mixed-design, Group (Lissajous, Color, and Tone) × Day (day 1, day 2, and day 3) × Session (the first, the second, and the last block of each day) analysis of variance (ANOVA) was conducted. The results showed that the three-way interaction did not reach significance, *F*(8,76) = 1.86, *MSE* = 7.45, *p* = .078, *η*^2^ = .01. Therefore, by conceptualizing learning speed as performance change per practice day (i.e., pairwise difference between RMSE of all blocks each day), the non-significant three-way interaction effect indicated that the gradient of improvement did not show any group difference on any particular day. Furthermore, the non-significant Group × Day interaction, *F*(4,38) = 1.56, *MSE* = 65.62, *p* = .204, *η*^2^ = .03, suggested that participants’ performance change between days did not differ among groups; the non-significant Group × Session interaction, *F*(4,38) = .73, *MSE* = 10.42, *p* = .577, *η*^2^ = .003, suggested that participants’ performance change across sessions did not differ among groups, either. However, the Day × Session interaction was significant, *F*(4,76) = 17.88, *MSE* = 7.45, *p* < .001, *η*^2^ = .05. Simple main effect analyses (collapsing across groups) indicated that all groups of participants significantly improved (RMSE reduced) on day 1 (mean difference of the first and the last blocks = -10.44 degrees, *p* < .001) and day 2 (mean difference of the first and the last blocks = -5.07 degrees, *p* < .001), but reached performance plateau on day 3 (mean difference of the first and the last blocks = -1.26 degrees, *p* = .183). Main effect for Group was also significant, *F*(2,19) = 26.25, *MSE* = 334.96, *p* < .001, *η*^2^ = .05. *Post hoc* analyses showed that Lissajous group performed better on average (lower RMSE) as compared with Color and Tone groups (both *p*s < .001), while the difference between Color and Tone groups did not reach significance (*p* = 1.000). Note that further investigation showed enlarged “Color vs. Tone” group difference at the late stage of learning (mean difference = 7.94 degrees, *p* = .089 in the last block of day 3, compared with mean difference = 1.19 degrees, *p* = 1.000 in the last block of day 2), suggesting that the Color group seemed to reach performance plateau and cease improving earlier than the Tone group.

### Performance in no-feedback transfer tests

**Fig 6:**
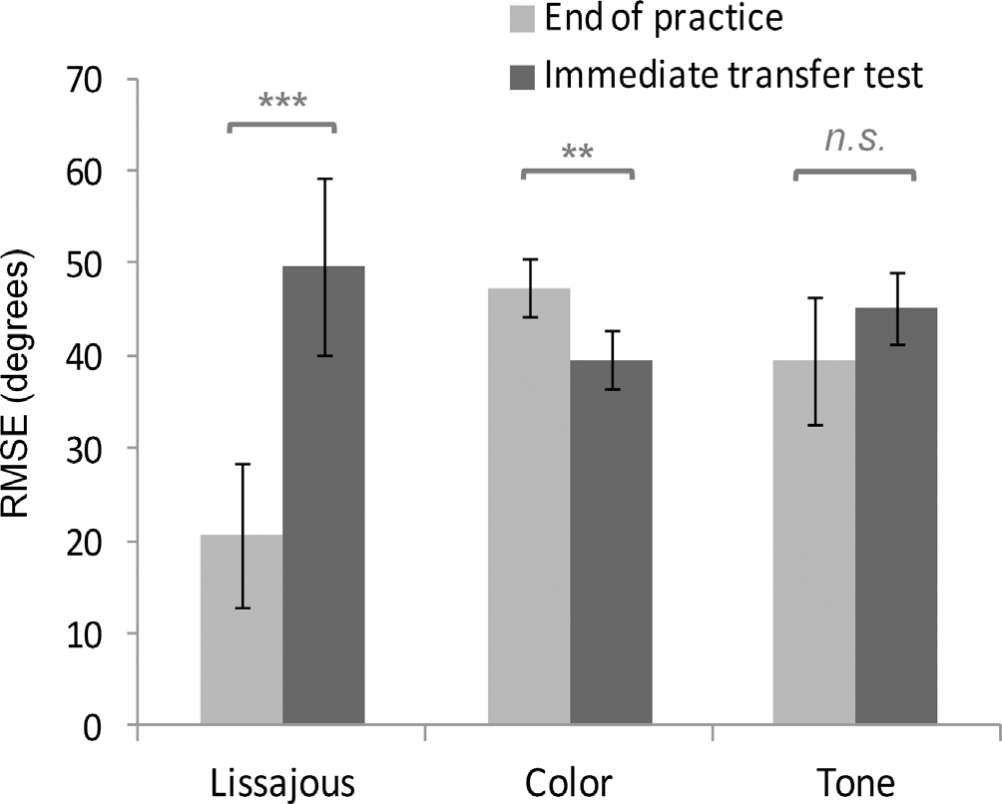
Performance change after feedback removal.

To examine whether different types of augmented feedback applied during the practice phase would result in different performance change upon their removal, a Group × feedback availability (with and without augmented feedback) mixed-design ANOVA was conducted on the RMSE of the last block of practice and the immediate no-feedback transfer test. Main effects for Group, *F*(2,19) = 5.54, *MSE* = 55.20, *p* = .013, *η*^2^ = .01, feedback availability, *F*(1,19) = 33.42, *MSE* = 26.33, *p* < .001, *η*^2^ = .22, and the Group × feedback availability interaction *F*(2,19) = 49.66, *MSE* = 26.33, *p* < .001, *η*^2^ = .66, were all significant. Simple main effect of feedback availability demonstrated significant performance deterioration in the Lissajous group, with mean difference of 29.00 degrees, *t*(7) = 8.48, *p* < .001, but not in the Tone group, mean difference = 5.71 degrees, *t*(6) = 2.24, *p* = .067. Surprisingly, the results showed significant performance improvement in the Color group when the feedback was removed, mean difference = -7.81 degrees, *t*(6) = -5.97, *p* = .001 (Fig 6). Looking at the interaction in the other way, *post hoc* comparisons also showed that while the Lissajous group outperformed the other two groups at the end of practice (both *p*s < .001), they performed significantly worse than the Color group (*p* = .021), and did not significantly differ from the Tone group (*p* = .571) in the immediate transfer test. The Color group did not differ from the Tone group either at the end of practice (*p* = .089) or in the immediate no-feedback condition (*p* = .367).

In addition to the immediate no-feedback transfer test, retention performance has also been taken as a key indicator of learning. Thus, a two-way Group × retention status (immediate vs. 24-hour retention) mixed-design ANOVA was conducted to evaluate the sustainability of performance after feedback removal. Retention status failed to reach significance, *F*(1,19) = .06, *MSE* = 15.28, *p* = .810, *η*^2^ = .003, demonstrating a fairly stable performance for all participants under different feedback conditions. Group difference was significant, *F*(2,19) = 7.85, *MSE* = 56.55, *p* = .003, *η*^2^ = .01, and subsequent *post hoc* comparisons showed that the overall performance of the Lissajous group was significantly worse than the Color group (*p* = .003), while other comparisons did not reach significance (*p* = .095 for Lissajous vs. Tone groups, and *p* = .411 for Color vs. Tone groups). Group × retention status interaction was not significant, *F*(2,19) = .85, *MSE* = 15.28, *p* = .445, *η*^2^ = .08.

### Performance in dual-task interference session

In order to examine the level of conscious control and the potential strategies adopted in the motor execution process, irregular rhythm counting was applied as a secondary task during the interference session, and participants’ performance was compared between dual-task and single-task conditions. RMSE was analyzed with a Group × interference condition (no interference, VR-C, VR-D, and AR-D) mixed-design ANOVA, where the 24-hour retention block on day 4 was taken as the single-task condition (no interference) in the analysis. Note that for single-task condition, although each trial extended for 40 seconds, only the first 25-second data was extracted for fair comparison with dual-task condition where each trial lasted for only 25 seconds. The first five seconds of each 25-second trial were also discarded to eliminate records of the movement initiation.

**Fig 7:**
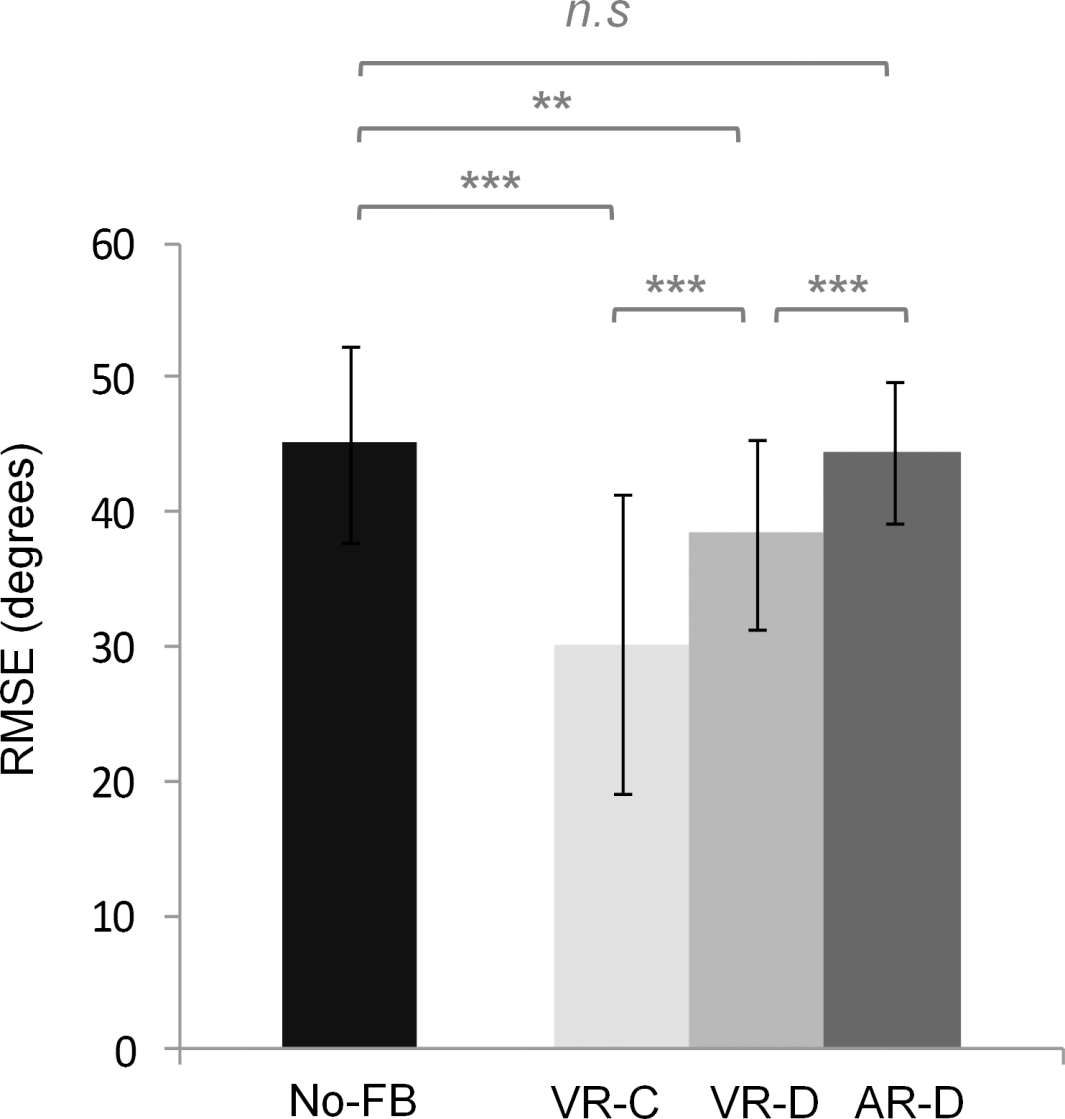
Motor performance in dual-task interference session. No interference (No-FB) indicated single-task condition before the interference began. Three different interference conditions are visual rhythm–continuous (VR-C), visual rhythm–discrete (VR-D), and auditory rhythm–discrete (AR-D).

The results showed significant main effect for interference condition, *F*(3,57) = 38.90, *MSE* = 26.94, *p* < .001, *η*^2^ = .63, but not for Group, *F*(2,19) = 2.98, *MSE* = 140.77, *p* = .075, *η*^2^ = .01. Group × interference condition interaction was not significant, either, *F*(6,57) = 2.13, *MSE* = 26.94, *p* = .064, *η*^2^ = .07. *Post hoc* comparisons on the interference main effect showed that, compared to the no interference condition (44.74 degrees), motor performance was significantly better in VR-C (29.91 degrees) and VR-D (38.36 degrees) conditions, *p* < .001 and *p* = .010, respectively, but not in AR-D condition (44.28 degrees), *p* = 1.000. When motor performances under different interference conditions were compared, the results showed significant differences among all three conditions. That is, VR-C was better than VR-D (*p* = .001) and AR-D (*p* < .001), and VR-D was better than AR-D (*p* < .001) (Fig 7).

Group means on counting accuracy in different interference conditions were all above 0.9, except for the Lissajous group in VR-C condition (mean accuracy = 0.83), demonstrating a fairly reasonable performance level overall. A mixed-design Group × interference type (VR-C, VR-D, or AR-D) ANOVA on counting accuracy showed that participants’ performance in the counting task was not significantly different among groups, *F*(2,19) = 1.74, *MSE* = .01, *p* = .203, *η*^2^ = .001, or the interference types, *F*(2,38) = 1.20, *MSE* = .01, *p* = .313, *η*^2^ = .05, while the Group × interference type interaction was marginally significant, *F*(4,38) = 2.68, *MSE* = .01, *p* = .046, *η*^2^ = .21. Subsequent simple main effect analysis indicated that the Group × interference type interaction was mainly due to lower accuracy in the Lissajous group in the VR-C condition compared with AR-D conditions, *p* = .041, which may imply a more severe dual-task interference to the Lissajous group in the VR-C condition, where the counting stimuli resemble more closely to the augmented feedback than in other conditions. No such difference was found in the other feedback groups.

## Discussion

The current study aimed to examine whether the differential “guidance effect” [1, 2] between feedback modalities [17] is a consequence of modality dominance or structural feature of the feedback information. In line with previous findings, our results showed that the bimanual coordination learning with Lissajous feedback was vulnerable to the feedback removal, indicating a strong dependence on the feedback, while learning with auditory feedback (Tone group) seemed to be more perseverant as participants’ performance sustained when the feedback was removed. Our results further demonstrated that "visual dominance” [18] is not a determining factor of inducing guidance effect since participants in the Color (visual rhythm) group did not show performance deterioration as the Lissajous group, but having sustainable performance in the test phase just like the Tone (auditory rhythm) group. Furthermore, the learning curve of the Color group appeared to resemble that of the Tone group rather than the Lissajous group, indicating that motor learning measured by the pattern of error reduction depends more on the structural feature of information provided by the augmented feedback.

Maslovat et al. [30] addressed a similar question of how the information types (continuous vs. discrete) of concurrent augmented feedback influence bimanual coordination learning by examining the effects of continuous Lissajous feedback and discrete visual feedback in the same 90°-out-of-phase bimanual coordination learning. Comparable to the current results, the study demonstrated superior performance in the Lissajous group during acquisition but inferior retention after feedback removal as compared to the discrete visual group. However, there are a couple of differences between the experimental design of Maslovat et al. [30] and the current study. First of all, the task design in the former study was more like a “tracking” or “synchronization” movement rather than a purely “self-generated” or “self-paced” movement, as the circular Lissajous template and the equally spaced metronome pulses were both provided with the movement. But in the current study, the targeted trajectory or rhythm was displayed prior to practice, and only augmented feedback was available during the motor execution process. Secondly, in the former study, discrete visual feedback was presented at each metronome pulse, illustrating participant’s arm location at that moment on two side-by-side linear templates. Therefore, the discrete visual feedback used in Maslovat et al. [30] was not a unified structure in terms of information processing, as two streams of motor information were represented by two separated visual feedback. On the contrary, discrete visual feedback in the current study integrated information from two effectors into one series of visual rhythm, which displayed in the center of the screen and was more comparable to the continuous Lissajous feedback.

Although the current results did not support the visual dominance account for the guidance effect, some modality-specific characteristics may still influence feedback processing. Given the same level of performance requirement (0.8Hz), the Color group in the current study seemed to reach performance plateau and cease improving earlier than the Tone group. The trend of diverged performance between the Color and the Tone group at the late stage of learning may reflect different modality advantages in processing rhythm information. We suspect that differences in temporal resolutions of auditory [20–23] and visual information [24] processing may contribute to the observed divergence at the late stage of learning. However, further studies are needed to verify this speculation.

In addition, due to the slow transduction process in the visual system, which is several tens of milliseconds slower than in the auditory system [31], the perception of the same rhythm information presented in visual and auditory modalities may differ significantly. It is likely that the transduction property makes rhythmic feedback in auditory format a more accurate source of information than visual format for matching arm movements. The asynchrony between feedback perception and arm movements may induce interference, especially to the Color group, and that may explain why the performance of the Color group improved significantly when the feedback was removed.

What type of information does the augmented feedback provide? Lissajous plot has been demonstrated to be effective in boosting bimanual coordination performance [10, 32–35] as it provides clear, integrated, and temporally continuous information about the arms’ movement. Rhythmic feedback, on the other hand, may be less reliable for guiding the movement as it provides only discrete information about the reversal points rather than a continuous mapping indicating instantaneous phase relationship between the two effectors. This challenging learning condition may encourage participants to intensively process the intrinsic sensory information, especially the proprioception, and thus effectively reduce the reliance on the augmented feedback by forming an independent motor representation which can be relied on after feedback removal.

Furthermore, another possible advantage of the rhythm information over the Lissajous figure is that, unlike the latter which maps information of the arm position to the 2-D Cartesian coordinates and is less intuitive to link back to the exact arm movements, the rhythmic feedback is more directly linked to critical features of the movement kinematics, i.e., the reversal points in the current study. Thus, by memorizing the target rhythm and its relation to the required movements during practice, participants can recall the rhythm later for “guiding” the movement when the feedback was removed. It might also be easier for the Color and Tone groups than the Lissajous group to convert this cyclical movement into verbal coding given the rhythmic nature of the augmented feedback, which in turn facilitates the rhythm recall process and probably encourage a more conscious way of control.

One surprising finding in the current study is the significant performance improvement rather than deterioration in all three groups under dual-task visual rhythm counting conditions (VR-C and VR-D) and no significant performance change in auditory rhythm counting condition (AR-D). That is, instead of interfering with the movement, the application of the irregular rhythm counting task improved the bimanual coordination. We speculate that the benefits from the combination of irregular rhythm counting task and the motor task may directly result from reduced cognitive resources for consciously controlling the movement. This relatively “unconscious” mode of control forced participants to rely on proprioception and other “low-level” sensory representation for performing the task. Hence, these results suggested that lower involvement of conscious control may benefit bimanual coordination, probably due to the greater involvement of proprioception in the control process.

Unlike the prediction by the guidance hypothesis, the current results suggested that even practicing with augmented feedback, the processing of intrinsic sensory information was not fully blocked, and it gradually formed the permanent motor representation and made the performance under dual-task condition possible. Note that similar performance change under dual-task condition was also observed in the Lissajous group, indicating that the intrinsic information such as proprioception can still be processed to some extent under such a strong feedback-guided learning environment. However, this learning effect may not be observable in a single-task no-feedback condition given that the feedback removal may shift participants’ attention from external (the augmented feedback) to internal (the arms’ movement) focus, and further trigger a conscious type of control which suppressed the involvement of proprioception and interfered with automaticity [36–38]. It is especially the case for the Lissajous group as their movements were mostly guided by the external stimuli. On the contrary, Color and Tone groups adopted a mixture of stimulus-guided and self-generated movement strategy and thus can preserve similar control as in the practice phase when augmented feedback was removed. Note that if the participants performed the coordination task in a fully autonomous way, we should see neither positive nor negative modulation of performance by a secondary cognitive task. Thus, the positive effects observed in the current study implied that certain level of conscious control may still be involved.

Moreover, the results showed that participants’ performance only improved in visual rhythm counting conditions (VR-C and VR-D) but not in auditory rhythm counting condition (AR-D). There are two possible explanations: First, although the irregular rhythm was generated in the same manner, and the counting difficulty was controlled across conditions, the cognitive load of performing counting task displayed through different modality channels may still be different. As mentioned above, given the better temporal resolution of auditory perception, the cognitive load of performing the auditory rhythm counting task might be lower than that of performing the visual rhythm counting task, and thus the secondary auditory rhythm counting task had less influence on the existing control process. The other possibility is that, if the rhythm counting task presented by visual and auditory inputs brought in ignorable difference in cognitive load, the observed performance difference might be attributed to stronger linkage between motor and auditory stimuli, especially in the temporal domain [39–43]. It made the irregular rhythm processing via auditory channel interfered more with the planned (cyclical and rhythmic) motor output than via visual channel.

With regard to the significant difference in performance between Lissajous and the other two groups at the beginning of learning (block 1), it may be intuitively attributed to individual difference or distinct level of difficulty when learning with different types of feedback. However, if that is the case, one would also expect distinct learning curves across groups. Specifically, if the Lissajous group by default had motor learning abilities superior to the other groups, their gradient of the learning curve would be steeper and reach performance plateau earlier. Likewise, if the initial difference was due to lower difficulty level for the Lissajous group, one would not observe continuous improvement as practice proceeded because the ceiling of performance should be reached pretty soon after a short period of practice. In fact, the gradient of improvement as a function of time did not show significant difference among groups, which means that the learning speeds were comparable across the three groups throughout the whole practice phase though different retention performance indicated that the exact skills learned by the Lissajous group may not be the same as the others. It is possible that the participants in the Lissajous group had only learned to use the feedback information more efficiently to generate accurate movement rather than to form an intrinsic, long-lasting control strategy. The intercept discrepancy of the learning curves, in our view, was mainly due to the initial advantage of learning the 90°-out-of-phase coordination pattern with Lissajous feedback given its intuitive and continuous representation of the relative phase.

In the current study, we have compared modality difference under discrete rhythmic feedback (Color vs. Tone groups) as well as different information types under the same visual modality (Lissajous vs. Color groups). To thoroughly verify the potential dissociation between feedback modality and information type, ideally an additional group that learns the bimanual coordination task with some sort of “continuous audiospatial feedback” could have been included to constitute a full factorial design. For example, it would be optimal to include an audio version of the Lissajous feedback for fair comparison. However, practical constraints on the design and the effectiveness of augmented feedback need to be carefully considered here. It is dubious whether a “sonified” Lissajous feedback can preserve comparable spatial precision of movement trajectory to its visual counterpart. The challenge is both biological and technical: It is speculated that the sonified Lissajous feedback would be perceived less precisely than the visual version due to lower spatial resolution of the auditory system than the visual system [44], which makes it not that useful for online movement corrections; technically, it is also quite expensive to present auditory information with high spatial precision. Therefore, the interpretability and the effectiveness of the sonified Lissajous feedback have yet to be evaluated. For future work addressing the interaction between the modality and information type of augmented feedback, it would be helpful to have a proper design of the continuous auditory feedback that is both cost-effective and conveys spatial information as precise and accurate as its visual counterpart.

## Conclusions

The present findings demonstrated that the dominant nature of visual modality is not a determining factor of inducing guidance effect, and the learning process as well as the retention performance depend more on the type of information that the augment feedback provided during practice. The study also showed potential benefits from dual-task conditions where the visual rhythm counting task was applied as a secondary task, indicating that lower involvement of conscious control may result in better performance in bimanual coordination. The findings are insightful for both understanding the essential factors contributing to motor learning and pedagogical purposes: Although augmented feedback has been widely adopted in the process of motor learning, what is the most effective way to assist motor learning still remains controversial. The current findings have shed some light on the theoretical perspectives as well as practical applications of the design and the usage of augmented feedback, and provided clues regarding how to benefit from augmented feedback while preventing potential dependence.

## Supporting Information

S1 Video Demonstration of the in-phase movement. (MPG)

S2 Video Demonstration of the anti-phase movement. (MPG)

S3 Video Demonstration of the 90°-out-of-phase movement. (MPG)
